# Meeting Abstracts from the 8th Annual Atlantic Corridor Medical Student Research Conference

**DOI:** 10.1186/s12919-023-00253-2

**Published:** 2023-05-18

## O1 Anaphylaxis management and adrenaline auto injector use: assessing knowledge and piloting a virtual educational intervention for parents of children with food allergies

### Cronin C.^1^, Keohane H.^1^, Flores Villarta L.^1^, O'Rourke E.^1^, Gallagher A.^2^ Tobin C.^2^ Velasco R.^3^, Trujillo W.^1,2^

#### ^1^Department of Paediatrics and Child Health, University College Cork; ^2^Department of Paediatrics, Cork University Hospital; ^3^Hospital Universitario Rio Hortega, Department of Paediatrics, Valladolid, Spain

*BMC Proceedings 2023*, **17(Suppl 4)**:O1

**Background**

Intramuscular adrenaline with an adrenaline auto injector (AAI) is the first-line therapy for anaphylaxis. However, many patients and caregivers are often unable to demonstrate correct administration of the AAI.

The aim of this study is to assess the knowledge of anaphylaxis management and adrenaline auto injector use among caregivers of children with food allergies and to pilot a virtual educational intervention among parents.

**Materials and Methods**

Parents of children with food allergies attending the paediatric allergy clinic at Cork University Hospital who were prescribed an AAI completed an online questionnaire regarding anaphylaxis management and were assessed in the use of their child’s AAI via a video call. A pilot group of parents without previous AAI training were invited to take part in a virtual educational intervention.

**Results**

Parents had good overall knowledge of the definition of anaphylaxis (91%), the management of allergic reactions involving the skin (92.1%) and the management of anaphylaxis with respiratory symptoms (86.5%). However, parents were less informed of the management of cardiovascular and gastrointestinal symptoms of anaphylaxis (50.2%). 20 (18%) parents correctly explained all 5 steps essential for successful AAI administration. All 10 parents who took part in the pilot study were satisfied with the educational intervention and knowledge was above baseline on reassessment.

**Conclusion**

This is the first study in Ireland to assess caregiver knowledge of anaphylaxis management and AAI administration. Parents had a good overall knowledge of anaphylaxis management. Virtual training for parents is an effective and safe method of anaphylaxis management training.

## O2 Investigating Endothelin-1 expression and transfer in breast cancer

### Mammen E.^1^, Dwyer R.M.^2,3^, O’Neill C.P.^2^, McCarthy E.C.^2^, Sugrue B.^2^, Chabria Y.^2,3^

#### ^1^University of Galway; ^2^Discipline of Surgery, Lambe Institute for Translational Research, University of Galway, Galway, Ireland; ^3^CÚRAM, the SFI Research Centre for Medical Devices, University of Galway, H91 W2TY Galway, Ireland

*BMC Proceedings 2023*, **17(Suppl 4)**:O2

**Background**

Patients with late-stage breast cancer (BC) face a poor prognosis; <30% surviving 5 years. Understanding mechanisms underlying preferential dissemination to bone could stratify high risk patients. Extracellular Vesicles (EVs) released by cells play an intrinsic role in shuttling proteins for cell communication. Potent vasoconstrictor, Endothelin-1 (ET-1), stimulates ossification and osteoblastic metastasis. This study investigated ET-1 expression in BC cells and plasma EVs to determine potential transfer in support of metastasis.

**Materials and Methods**

Different epithelial BC cell subtypes were cultured: T47D, BT-474, SK-Br-3, HCC-1954 and MDA-MB-231. EVs were isolated from healthy premenopausal volunteers’ plasma (n=6) through Size Exclusion Chromatography. EV size distribution and concentration were characterised by Nanoparticle Tracking Analysis. RNA was extracted from cells and EV isolates using MagNA Pure Compact. RQ-PCR targeted ET-1 and endogenous control, Peptidylprolyl-Isomerase-A (PPIA).

**Results**

Successful RNA extraction provided a high yield of quality RNA from cell pellets (~1μg/μL yield, A260/280nm ratio of 2), while EV yield was low. PPIA was stably expressed across all cells (<2 Cycle Threshold (CT) variation). ET-1 expression was robust in all cell lines; strongest in Sk-Br-3 (CT 23.8) and weakest in HCC-1954 cells (CT 29.6). Plasma EV isolation revealed the appropriate size of EVs (<200nm). Fractions 1-3 exhibited optimal EV size distribution (89.9-126.9nm). EV concentration ranged from: 1.53x108-1.01x109 particles/mL. ET-1 was not detected in EVs.

**Conclusions**

ET-1 mRNA expression was robust in all BC cells but not in EVs of healthy volunteers. Further investigation to determine ET-1 expression in EVs of BC patients and protein level transfer is warranted.

## O3 Why magnetic resonance imaging is mandatory in patients presenting with first seizures: a diagnostic yield of first-line investigations

### O'Donoghue C.^1^, Chaila E.^2^, O’Reilly E.^1^, Costello D.^1^

#### ^1^University College Cork; ^2^University Hospital Limerick, Ireland

*BMC Proceedings 2023*, **17(Suppl 4)**:O3

**Background**

Recent revision of the International League Against Epilepsy’s operational definition of Epilepsy allows a diagnosis to be made after a single seizure with a ≥60% chance of experiencing another in the future. Consequently, detection of epileptogenic lesions on structural brain imaging is important. The difference in diagnostic yield from low resolution computed tomography (CT) compared to a high resolution magnetic resonance imaging (MRI) has not been quantified in a population-based cohort study.

**Materials and Methods**

Using multiple overlapping methods of case ascertainment and classification by an epileptologist, all patients who presented with a first seizure (n = 1330) were identified in a defined geographical area (Cork City and County Cork, Ireland; population 550,000) from January 1, 2017 to December 31, 2017. Three cohorts were defined: new onset epilepsy, unprovoked first seizures, provoked first seizures. CT and MRI results were evaluated.

**Results**

When both CT and MRI were performed (n = 127), 24.41% (n = 31) of patients had MRI detection of epileptogenic lesions not evident on CT (p = 0.0013). New epilepsy criteria had significantly higher MRI abnormalities than the previous definition (p = 0.0036) and single seizure low risk (p < 0.0001). Focal seizures had significantly higher yield of epileptogenic lesions on MRI imaging than generalized (p < 0.0001).

**Conclusion**

First presentation seizures warrant MRI brain imaging as epileptogenic lesions were not detected in a significant number of patients by CT who were detected by MRI. Furthermore, focal seizures are more associated with epileptogenic lesions on brain imaging than generalized.

## O4 Effect of treatment of gestational diabetes on pregnancy outcomes – a retrospective study

### Kazemian R.^1^, Bogdanet D.^2^, Trulea A.^3^

#### ^1^School of Medicine, University of Galway, Galway, Ireland; ^2^Department of Diabetes and Endocrinology, Mayo University Hospital, Castlebar, Mayo, Ireland; ^3^Department of Gynecology and Obstetrics, Mayo University Hospital, Castlebar, Mayo, Ireland

*BMC Proceedings 2023*, **17(Suppl 4)**:O4

**Background**

Gestational diabetes mellitus (GDM) is associated with an increased risk of adverse pregnancy outcomes. Treatment of GDM has been shown to lead to a reduction in hyperglycaemia-related pregnancy complications. Data on the impact of each type of intervention on pregnancy outcomes is limited. The aim of this study was to assess if women with GDM treated with diet and exercise (GDM-DE), metformin (GDM-M) and insulin (GDM-I) have comparable outcomes.

**Materials and Methods**

This retrospective cohort study included 277 singleton pregnant women with GDM (144 GDM-DE, 48 GDM-M and 85 GDM-I) at Mayo University Hospital, Castlebar, Ireland between 2020 and 2021. Maternal and foetal outcomes were recorded and analyzed using Pearson’s Chi Square and odds ratio. A p-value of <0.05 was considered to be significant.

**Results**

The difference in percentages for having a postpartum haemorrhage (PPH), compared to GDM-I, was 10.4% higher for GDM-M and 2.1% higher for GDM-DE (p=0.004). Despite treatment type, there was no difference between the three groups for maternal outcomes including spontaneous onset of labour, induction of labour, spontaneous vaginal delivery, instrumental delivery, emergency caesarean, perineal tear, episiotomy and polyhydramnios. Additionally, there was no difference between the three groups for neonatal outcomes including preterm delivery, large for gestational age, small for gestational age, macrosomia, Apgar score, hypoglycemia and neonatal intensive care unit admission.

**Conclusion**

GDM-M was associated with higher PPH rates compared to GDM-I and GDM-DE. This highlights the need for more research to analyze the association between metformin and adverse pregnancy outcomes in mothers with GDM.

## O5 Sexual health knowledge and behaviour within the Irish gay, bisexual & men-who-have-sex-with-men population: a latent class analysis

### Tavolieri M.^1^, Davoren M.^2^

#### ^1^University College Cork; ^2^Sexual Health Centre Cork

*BMC Proceedings 2023*, **17(Suppl 4)**:O5

**Background**

Despite the many health initiatives aimed at reducing sexually transmitted disease (STI) transmission, the rates of STIs amongst the men-who-have-sex-with-men (MSM) remains disproportionally high. In Ireland for example, despite the MSM population making up approximately 6% of the population, they accounted for 49% of new HIV infections in 2019. Past research has shown that certain factors - alcohol use, for example - play a significant role in determining whether individuals make safe sexual health choices. These studies, however, tend to group MSM populations by geographic area (ex. Country, City), ignoring possible subgroups.

**Materials and Methods**

Here we’ve evaluated the Irish respondents’ data set from the European MSM Internet Survey (EMIS) 2017 for distinct classes of respondents. We then identified four exogenous variables which contribute to class membership.

The EMIS 2017 was a Europe-wide online self-report survey with over 400 questions relating to sexual health behaviours, knowledge, and other lifestyle aspects. Latent class analysis (LCA) was employed to identify distinct classes of respondents. Multivariate analysis was used to determine correlation between several exogenous variables and class membership.

**Results**

From this analysis two classes emerged: one characterized by high knowledge and compliance with recommendations and the other with low. Membership in the lower knowledge/compliance group was significantly affected by level of education, sexual orientation, “outness” and level of internalized homonegativity (IH).

**Conclusion**

These findings suggest that future health initiatives would benefit from targeting individuals of lower education, those who do not identify as gay, who are “out” to very few people, or who live with high levels of homonegativity.

## O6 Radiographic and clinical outcomes of Attune and Triathlon total knee arthroplasty systems

### O'Donovan P.^1^, McAleese T.^2^, Harty J.^3^

#### ^1^University College Cork; ^2^South Infirmary Victoria University Hospital; ^3^Department of Surgery UCC/SIVUH

*BMC Proceedings 2023*, **17(Suppl 4)**:O6

**Background**

**I**Total Knee Arthroplasty (TKA) is well established for improving pain and function. The Attune system was introduced in 2013. However, reports suggest early tibial debonding. Here we compare the incidence of radiolucent lines (RLLs), survival and patient reported outcome-measures (PROMs) of the Attune with the Triathlon.

**Materials and Methods**

All Attune (N=445) and Triathlon (N=285) TKAs implanted in 2015 and 2016 at SIVUH were reviewed. RLLs were documented using the Modern Knee Society Radiographic Evaluation System. Five year survival was assessed using Kaplan-Meier analysis with Log Rank method for significance. PROMs were collected pre-op, 6 months, 2 years and 5 years post-op and compared using the Kruskal-Wallis Test.

**Results**

The same incidence RLLs between Attune and Triathlon (17.75%, 60/338 vs 17.75%, 41/231; p=0.49). Attune had higher proportion in tibia [87.1%(54/62) vs 54.5%(27/44); p= 0.001] and implant-cement interface [62.9%(39/62) vs 43.18%(19/44); p=0.02]. Conversely, the Triathlon had higher proportion in femur [38.64%(17/44) vs 12.9%(8/62); p=0.001] and cement-bone interface [56.82%(25/44) vs 37.1%(23/62); p=0.02]. No difference in revision-free survival (Attune 97.8% vs Triathlon 95.8%; p=0.129). Attune performed better at 5 years in OKS [Attune=42.57 (SD 5.24) vs Triathlon=41.03 (SD 6.35); p=0.001] and EQ-5D [Attune=0.773 (SD 0.187) vs Triathlon=0.729 (SD 0.218); p=0.013]. No difference at 5 years in EQ-VAS [Attune=80.37 (SD 13.67) vs Triathlon=78.53 (SD 15.27); p=0.25].

**Conclusion**

Similar incidence of RLL and survivorship at 5-years between Attune and Triathlon. Improvements in PROMs modestly favour the Attune. These results contrast with some alarming reports of early tibial failure in the Attune system.

## O7 Monoclonal Gammopathy of Renal Significance in the West of Ireland – Adherence to diagnostic and management criteria: a retrospective clinical audit

### Forde V.^1^, McHale T.^2^, Kiernan N.^3^, Krawczyk J.^4^

#### ^1^University of Galway; ^2^Department of Pathology, University Hospital Galway, Galway, Ireland; ^3^Department of Nephrology, University Hospital Galway, Galway, Ireland; ^4^Department of Haematology, University Hospital Galway, Galway, Ireland

*BMC Proceedings 2023*, **17(Suppl 4)**:O7

**Background**

Monoclonal Gammopathy of Renal Significance (MGRS) describes a group of disorders involving kidney damage caused by monoclonal immunoglobulins produced by a non- or pre-malignant plasma cell or B-cell clone. If untreated, they are likely to result in end-stage renal failure. The aim of this audit was to establish adherence to the diagnostic criteria and workup of MGRS patients, as set out by the 2018 Expert Consensus Document of the International Kidney and Monoclonal Gammopathy Research Group.

**Materials and Methods**

All patients who underwent a renal biopsy in University Hospital Galway from January 2017 to June 2022 (n=464) were included. Data were collected retrospectively from histology reports, letters and laboratory results and analysed using Microsoft Excel and SPSS 26. Audit approval was granted from GUH Clinical Audit Committee.

**Results**

28 patients (17 females, 11 males), mean age 55.5 ± 15.7 years, were identified with possible MGRS- or myeloma-associated renal lesions, with 2 excluded due to data unavailability. 85% (22/26) had serum and/or urine protein electrophoresis and immunofixation performed. There was a trend towards greater compliance of monoclonal immunoglobulin testing from 2018-2022, with full compliance seen in the last 3 years. After paraprotein workup, 58% (15/26) were considered to have MGRS- or myeloma-related lesions.

**Conclusion**

It is important that all patients with possible MGRS-associated renal lesions undergo monoclonal immunoglobulin testing. As monoclonal immunoglobulin deposits are minimal in C3 glomerulopathy and thrombotic microangiopathy, further serological testing should be undertaken to rule out MGRS.

**Disclosure**

*Funding from School of Medicine, University of Galway.*

## O8 Awareness of modifiable lifestyle risk factors and acceptability of secondary risk reduction services amongst Irish breast cancer survivors and oncology clinicians

### Steele C.^1^, O'Reilly S.^2^

#### ^1^University College Cork; ^2^Department of Medical Oncology, Cork University Hospital, Ireland, and Cancer Research @UCC, College of Medicine and Health, University College Cork, Ireland

*BMC Proceedings 2023*, **17(Suppl 4)**:O8

**Background**

Many patients (>75%) diagnosed with breast cancer in Ireland today will be cured and are at risk of dying from other cancers and non-communicable diseases. Breast cancer diagnosis provides a pivotal time point for education on modifiable risk factors and engagement with secondary risk reduction services. Our study set out to establish the level of awareness and acceptability of these areas, respectively.

**Materials and Methods**

A survey was developed for breast cancer survivors and oncology clinicians using previously validated questionnaires; the ‘Mitchelstown Cohort Survey’ and the ‘International Physical Activity Questionnaire’.

**Results**

Between September and December 2021, 322 patients and 29 clinicians attending the South Infirmary Victoria and Cork University Hospitals, completed the survey. Over 75% of patients and clinicians were aware of modifiable cancer risk factors. 90% clinicians were willing to refer to services however only 1 in 5 had training in secondary risk reduction. Patients who smoked, had increased alcohol intake, or gained weight since diagnosis were more likely to engage with services (p=<.001, p=<.001, p=0.015 respectively). Education level had an impact on the likelihood of patient engagement. Patients who increased activity levels since diagnosis were more likely to engage with exercise education (p = 0.015).

**Conclusion**

This study identified that 75% of breast cancer survivors and oncology clinicians were aware of the importance of modifiable risk factors. While clinicians were willing to refer to services, the likelihood of patient engagement was associated with their ‘at risk’ behaviours and education level. Our study highlights the challenges of implementing health promotion programs in this cohort.

## O9 Oral colonisation by drug resistant Gram-negative bacteria among patients with a diagnosis of treatment resistant schizophrenia

### Mangalam Lonappan, A.^1^, McDonagh, F.^1^, Miliotis, G.^1^, Kumar Singh, N.^2^, Venkateswaran, K.^2^, Hallahan, B.^3^, O' Connor, A.^1^, Mc Evoy, N.^1^

#### ^1^Antimicrobial Resistance and Microbial Ecology Group, School of Medicine, University of Galway, Galway, Ireland; ^2^Biotechnology and Planetary Protection Group, NASA Jet Propulsion Laboratory, California Institute of Technology, Pasadena, California, USA; ^3^Discipline of Psychiatry, School of Medicine, University of Galway, Galway, Ireland

*BMC Proceedings 2023*, **17(Suppl 4)**:O9

**Background**

Clozapine is an atypical antipsychotic utilised in the management of patients with treatment resistant schizophrenia (TRS). Chronic use has been reported to be linked with disturbances of the healthy microbiome allowing for colonisation with pathobionts. This colonisation leads to an increased risk of transmission as well as pulmonary pathologies. This study aimed to be the first to characterise the prevalence of oral colonisation with pathobionts in patients on clozapine.

**Materiald and Methods**

Saliva samples were collected from 45 patients on clozapine and plated on selective agar plates. Susceptibility to 16 antibiotics was defined. The DNA from all isolates was sequenced and sequencing data was processed bioinformatically to characterise the genetic ability for virulence and resistance. Clinical data was retrieved, and univariate statistical analysis was conducted to assess other factors affecting oral colonisation with pathobionts.

**Results**

51% of the patients on clozapine were found to be orally colonised with pathobionts. This compares to a colonisation rate of 5% reported in the general population. No other factor analysed appeared to affect the colonisation rate. Overall, 13 species were identified. Indications of the spread of a multidrug resistant, hypervirulent strain of Citrobacter koseri amongst the cohort were noted. Furthermore, we reported the first oral identification of a multidrug and hypervirulent strain of the novel human pathogen Kalamiella piersonii.

**Conclusion**

The observations of this study provides the first direct link between the use of atypical antipsychotics and an increased risk for oral colonisation with drug and virulent resistant pathobionts capable of establishing pulmonary infections

## O10 Combining the best of old and new: advanced tools in pathology teaching

### Gray N.^1^, Hayes S^.2^

#### ^1^University of Galway; ^2^Discipline of Pathology, University of Galway, Ireland Consultant Histopathologist, Dept of Anatomic Pathology, University Hospital, Galway

*BMC Proceedings 2023*, **17(Suppl 4)**:O10

**Background**

Historically, encased 'pots' containing gross pathological specimens collected from post-mortem examinations have formed an integral part of pathology teaching. However, the use of these specimens has fallen out of favour in recent years. Our aim is to reinvigorate the pots at the University of Galway through the introduction of novel tools, and to create templates which can be adopted and adapted by other disciplines. Quick Response (QR) codes are an incredibly useful tool, and the medical field has been slow to adopt their use. In this study, we used QR codes to encourage students to interact with the pots and enhance their learning experience.

**Materials and Methods**

The study took place over ten weeks. We used the pots contained within the pathology museum at University Hospital Galway, focusing on those with central nervous system (CNS) pathology. CNS pathology was studied in detail, using past tutorials alongside online resources. Further information was collected from the pathology museum booklet which contained the diagnoses, clinical history, and gross findings of each specimen. Beaconstac was used to generate QR codes. Each QR code corresponds to between one and ten pots. Each QR code contained descriptions of the specimen with patient identifiers removed, followed by questions about the disease. Answers were provided with detailed descriptions and images to enhance understanding.

**Results**

We demonstrated that utilising QR codes is feasible in pathology education and that they have the potential to revitalise the use of pots at medical institutions. The codes can be scanned by students, making tutorials more interactive. The questions and answers within each QR code encourage retrieval practice and active learning, which enhances long-term retention of information.

**Conclusions**

In this project, we amalgamated old pathology teaching methods with new QR technology to illustrate that novel methods can be employed to maximise the utility of pots in pathology teaching. Our study has exciting implications, as it paves the way for future developments in medical education. Our hope is that utilising QR codes in this manner is an initial step towards introducing more advanced teaching tools, such augmented reality technology.

## O11 Coordination of healthcare services for adult survivors of childhood cancer in Ireland: a national qualitative study

### Mulcahy E., Barrett P.

#### University College Cork

*BMC Proceedings 2023*, **17(Suppl 4)**:O11

**Background**

Survivors of childhood cancer require comprehensive lifelong follow-up and holistic support beyond the acute cancer treatment phase. We sought to assess the reported experience, and coordination, of follow-up healthcare services for adult survivors of childhood cancer in Ireland.

**Methods**

This is a qualitative study based on secondary analysis of existing data (transcripts of seven focus groups conducted in 2018 with survivors of childhood cancer who were diagnosed before the age of 18 and their parents). Thirty-three participants were included (15 survivors and 18 parents).

**Results**

The following four themes were generated: (a) Suboptimal patient empowerment; many survivors weren’t provided with adequate education or information on the treatment they had received or their risk of late-effects. (b) Fragmented follow-up care; following completion of treatment patients were faced with a lack of regular medical follow-up, scattered services and a poorly organised transition from paediatric to adult care. (c) Barriers to accessing services; financial and geographical factors, along with a general lack of specialised survivorship resources, contributed to inadequate service provision. (d) Desired model of care; patients emphasised the need for a central liaison setting/late effects clinic and a single point of contact (e.g. advanced nurse practitioner) to communicate with them and with their healthcare providers and to coordinate their follow-up care.

**Conclusion**

Long-term follow-up services for adult survivors of childhood cancer in Ireland are fragmented and appear to lack overall coordination. This is likely to pose a growing challenge into the future as survival rates improve and this population expands.

## O12 Development of a co-culture system to quantify phagocytosis of apoptotic cells by human monocyte-derived macrophages

### Hontz S.^1^, Ou Q.^2^, Griffen M.^1,2^, Powers, Rachel^2^,

#### ^1^University of Galway, School of Medicine; ^2^REMEDI, University of Galway

*BMC Proceedings 2023*, **17(Suppl 4)**:O12

**Background**

Ex vivo-expanded regulatory T cells (T-reg) may modulate the phagocytic activity of macrophages. This study aimed to optimize conditions for quantifying the phagocytic potency of human monocyte-derived macrophage (MDM), with the eventual goal of testing how T-reg cells modify phagocytosis of MDM.

**Materials and Methods**

Apoptosis induction of Jurkat cells (JC) was optimised by exposure to UVB light (285nm) for 10-30min followed by culture for 2.5-4.5h. Apoptosis was detected by staining with Annexin V (apoptosis) and Draq7 (necrosis) and analysing by flow cytometry (Cytek NL3000). Apoptotic JC cell were defined as “Annexin V+/Draq7-”. To generate MDM, fresh human peripheral blood mononuclear cells (obtained via Ficoll centrifugation), were cultured in T175 flasks for 24h and then adherent cells (predominantly monocytes) were harvested and re-seeded in 6-well plates at 1.5 million/well with 20ng/ml of the macrophage growth factor GM-CSF. At day 9, the differentiated MDM were co-cultured with apoptotic JC (ratio of 1:5) that had been fluorescently labelled with Celltracker-CM-DiI. Co-culture was performed at either 4 or 37oC for 20 or 45min, after which the cells were lifted, stained with anti-CD11b Per-CP and analysed by flow cytometry. Phagocytotic MDM were defined as “CD11b+/CM-Dilhi”.

**Results**

Exposure of JC to UVB for 15min induced the highest early apoptosis rate (52.1%) followed by 10min (47.8%) and 30min (20.8%). The optimal post-exposure culture time was found to be 3-3.5h. Shorter or longer UVB exposure and culture times time resulted in higher rates of live (AnnexinV-/Draq-) or necrotic (AnnexinV+/Draq7+) cells rather than apoptotic cells (< 5%). In a co-culture experiment of MDM and CM-DiI-JC, the proportions of phagocytotic MDM present were 30.5% after 45min and 9.1% after 20min. co-culture at 37°C; but were only 1.5% and 1.2% following co-culture for the same times at 4°C. Optimal conditions were identified for UBV-induced apoptosis in JC and for generation of MDM from primary human monocytes.

**Conclusion**

A co-culture protocol was successfully developed for quantifying temperature-dependent MDM phagocytosis under varying conditions by flow cytometry. In ongoing work in the laboratory, this protocol will be employed to investigate the modulating effects of human T-reg on macrophage phagocytosis of apoptotic cells.

## P1 Child abuse knowledge and reporting practices in Cork University Hospital

### Abdul Hamid H.H.B^1^, Finn D.^2^

#### ^1^University College Cork; ^2^Paediatrics and Child Health Department, University College Cork

*BMC Proceedings 2023*, **17(Suppl 4)**:P1

**Background**

1. To assess and compare knowledge of child abuse presentation and reporting procedures in CUH Paediatric and Emergency Department healthcare staff.
2. To review child abuse reporting practices by looking at reporting experience, attitudes towards reporting, and likelihood to report.
3. To identify any factors affecting knowledge level and reporting practices.

**Materials and Methods**

This is a cross-sectional study done by distributing online and physical questionnaire to healthcare staff in CUH Paediatric and Emergency department who were conveniently sampled. The questionnaire was developed based on Children First National Guidance 2017 and was pilot tested on 10 medical students. For the assessment of knowledge level, correct answers are scored 1 while wrong and unsure answers are scored 0. For reporting practices, a 5-point Likert score was used and a higher score indicates a more positive reporting practices.

**Results**

67 responses are included in the study. The overall mean score for knowledge of child abuse presentation and reporting procedure is 11.9 out of 15. In terms of reporting experience, 79.1% of respondents have seen a suspected child abuse case but only 47.8% have experience reporting. A higher knowledge level is statistically correlated with a more positive reporting practice but no factors were identified to affect them independently. There is also no significant difference found between the two departments.

**Conclusion**

Healthcare staff in CUH Paediatric and Emergency department have satisfactory level of knowledge and reporting practices. The level of experience, profession type, or Children First training did not affect knowledge or reporting practices.

## P2 In-vitro characterisation of human umbilical cord-derived mesenchymal stromal cells (hUC-MSCs) cultured on a decellularised scaffold under macromolecular crowding conditions (MMC)

### Aguh M.^1^, Sanz-Nogués C.^2^, Du S.^2^, Fagan L.^2^, O'Brien T.^2^

#### ^1^University of Galway; ^2^Regenerative Medicine institute (REMEDI), University of Galway, Ireland.

*BMC Proceedings 2023*, **17(Suppl 4)**:P2

**Background**

MSC delivery to the wound site, to aid in the healing process, is being looked at as an advanced therapy for non-healing diabetic foot ulcers. This is due to MSCs appealing immunomodulatory, anti-inflammatory and pro-regenerative properties. Biomaterials made of native or synthetic extracellular matrix (ECM) components have been used as carriers to deliver MSCs into the wound bed to help circumvent the issues caused by direct cell injections. MMC involve the addition of macromolecules to media to mimic the in-vivo environment and have been shown to accelerate ECM deposition. Our aim was to characterise the in-vitro properties of hUC-MSCs grown on a decellularised bidirectional porcine peritoneal membrane (XenoMEM™) under MMC.

**Materials and Methods**

1 million cells were seeded on the basement membrane (BM) and connective tissue (CT) sides of the scaffold and cultured with and without MMC for 4 days. In-vitro properties of hUC-MSCs were assessed including cell metabolic activity (PrestoBlue™), proliferation (Picogreen™), and viability (LIVE/DEAD®). Cell seeding efficiency and distribution was also assessed.

**Results**

Cells were shown to be viable and metabolically active after 4 days in culture and were found to not proliferate when seeded on the scaffolds. About 10% of cells were found to be lost during the seeding process. Cells were found to be localised to the side they were seeded onto, and no cell migration towards deeper layers of the scaffold was observed. Cells formed a compact multilayer when grown on the BM side, while cells on the CT side were more dispersed. Overall, results showed that the in-vitro properties of cells were not affected by the scaffold side they were seeded onto, or by the media conditions they were cultured in.

**Conclusion**

The proposed cell-scaffold bio-complex construct could be used as a potential therapeutic product for diabetic wound healing. Our results show that the in-vitro properties of hUC-MSCs were maintained when cultured on this scaffold. Nevertheless, further analysis needs to be conducted to confirm whether MMC enhance ECM deposition in cells grown on this scaffold. Finally, the therapeutic efficacy of this construct will be further assessed in a preclinical model of diabetic wound healing.

## P3 Design and construction of a GAP-43 reporter system for potential identification of effective therapeutics for nerve regeneration

### Carlos-De Clercq N., McCarthy T.

#### University College Cork

*BMC Proceedings 2023*, **17(Suppl 4)**:P3

**Background**

Peripheral nerve injury (PNI) is a condition that can result in muscle paralysis and sensory disturbances. Electrical stimulation and/or the application of exogenous neurotrophic factors and cytokines are effective at enhancing nerve regeneration and is mediated via the expression of regeneration associated genes (RAGs) such as growth associated protein (GAP-43). Therapeutics upregulating GAP-43 have potential use as treatments for improving recovery from PNI. Few studies have investigated the potential of increasing GAP-43 for PNI therapeutic purposes and current methods for measuring GAP-43 expression are limited. The broader aim of this work is to construct a motor neuron-like cell model with a GAP-43 reporter system. Such a model would have potential use in screening for novel therapeutics that upregulate GAP-43 and in the optimisation of electrical stimulation treatment in combination with these therapies.

**Materials and Methods**

The key aim of the work is to design and construct a Cas9 plasmid bearing a gRNA that targets GAP-43 cleavage and a donor plasmid bearing a reporter cassette with homology arms (HAs) for the insertion of the reporter immediately 3’ of the GAP-43 promotor via CRISPR/Cas9 homology directed repair (HDR). Construction of these two plasmids is key to enable quantitative measurement of endogenous expression of the GAP-43 gene.

**Results**

To guide the Cas9 nuclease to the target location, GAP-43 gRNA oligomers were designed and cloned downstream of the U6 promoter in the Cas9 expression plasmid px330, which also expresses the Cas9 gene and the cloned gRNA when transfected into cells.

Results: For CRISPR/Cas9 HDR, the 5’ and 3’ GAP-43 HAs were amplified from mouse genomic DNA and cloned donor plasmid so that they flanked the reporter cassette.

**Conclusion**

This work successfully constructed the Cas9 gRNA expressing plasmid to target cleavage of the GAP-43 gene in mouse cell lines and has provided the complete design and construction foundation for generating the reporter donor plasmid.

## P4 Abdominal aortic aneurysm artificial circulatory system for medical device testing: 3D reconstruction from CT scans

### Aversa S.^1,2^, Silva N. P.^3^, Elahi M. A.^3^

#### ^1^University of Galway; ^2^School of Medicine Surgery and Obstetrics, University of Galway; ^3^Translational Medical Device Lab, University College Hospital Galway, School of Engineering, University of Galway

*BMC Proceedings 2023*, **17(Suppl 4)**:P4

**Background**

Endovascular Aneurysm Repair (EVAR) is the main surgical treatment for Abdominal Aortic Aneurysms (AAA). Post-surgical complications include graft migration and endoleaks. To monitor for such complications, patients are required to undergo regular follow-up imaging surveillance. Studies have proposed to monitor graft functioning by using a chronically implantable device to avoid the need for radiological imaging surveillance. An implantable sensor for the post-EVAR surveillance of AAA - the Aortowatch - is being developed at the Translational Medical Device Lab, UG. This study has proposed 3D AAA latex models and an artificial circulatory system to be used as a test platform for the chronic implantable device, the Aortowatch.

**Materials and Methods**

In this study, the 3D Slicer software was used to convert the Computed Tomography images of real patients into 3D AAA models. The models were 3D printed to create the mould. The final latex model was created by dipping the mould in liquid latex. To create the artificial circulatory system, the pulsatile pump FlowTek125 was connected to the AAA model and distilled water was pumped through the system.

**Results**

The study was successful in recreating an anthropomorphic AAA artificial circulatory system. Three AAA models of different sizes were produced and successfully connected to the artificial circulatory system. These models can be used in the further development and research of chronic implantable devices.

**Conclusion**

The outcomes of this research provide sufficient evidence for the use of the AAA artificial circulatory system for experimental studies.

**Disclosure**

Funding from School of Medicine, UG.

## P5 Hypertensive disorders of pregnancy and long-term risk of maternal stroke: a systematic review and meta-analysis

### Brohan M.^1^, Kelly L.^2^, McCarthy F.P.^3^, Khashan A.S.^4,5^, Barrett P.M.^4^

#### ^1^School of Medicine, University College Cork, Cork, Ireland; ^2^Department of General Medicine, Beaumont Hospital, Dublin, Ireland; ^3^Irish Centre for Maternal & Child Health, University College Cork, Cork, Ireland; ^4^School of Public Health, University College Cork, Cork, Ireland; ^5^Kublickiene, Karolina, Division of Renal Medicine, Department of Clinical Intervention, Science and Technology, Karolinska Institutet, Stockholm, Sweden

*BMC Proceedings 2023*, **17(Suppl 4)**:P5

**Background**

Hypertensive disorders of pregnancy (HDP) are associated with long-term risk of cardiovascular disease in parous women in later life. However, relatively little is known about whether HDP are associated with an increased risk of ischaemic stroke (IS) or haemorrhagic stroke (HS) in later life. This systematic review aims to synthesize the available literature on the association between HDP and long-term risk of maternal stroke.

**Materials and Methods**

PubMed, Web of Science, and CINAHL were searched from inception to 1st June 2021. Papers included were case-control or cohort studies, conducted on human participants, available in English, measuring the exposure of a history of HDP (preeclampsia [PE], gestational hypertension [GH], chronic hypertension, or superimposed preeclampsia), and the outcome of maternal IS or HS. Three reviewers extracted data and appraised study quality following the Meta-analyses of Observational Studies in Epidemiology (MOOSE) guidelines and using the Newcastle-Ottawa scale. The primary outcome was any stroke (AS) and secondary outcomes included IS and HS. The review protocol was registered on PROSPERO (CRD42021254660).

**Results**

21 studies met the inclusion criteria. HDP were significantly associated with AS, aRR 1.76 (95% CI, 1.41-2.20). PE was significantly associated with AS, aRR 1.71 (95% CI, 1.50-1.96), IS, aRR 1.68 (95% CI, 1.17-2.43), and HS, aRR 2.45 (95% CI, 1.36-4.41), respectively. GH was significantly associated with AS, aRR 1.23 (95% CI, 1.20-1.26), and IS, aRR 1.38 (95% CI, 1.14-1.67).

**Conclusion**

In conclusion, exposure to HDP, including PE and GH, appears to be associated with an increased risk of AS and IS, in parous women in later life.

## P6 Tracking COVID-19 amongst Galway University Hospital healthcare workers

### Bhat P.^1^, Murphy D.^1,2^, Kernan M.^1,2^, Laffey J.^1,2^, Fleming C.^1,3^, McNicholas B.^1,2^

#### ^1^School of Medicine, University of Galway, Galway, Ireland; ^2^Department of Anesthesiology and Intensive Care Medicine, Galway University Hospital, Galway, Ireland; ^3^Department of Infectious Disease, Galway University Hospital, Galway, Ireland

*BMC Proceedings 2023*, **17(Suppl 4)**:P6

**Background**

When the COVID-19 virus emerged, the scientific community directed its efforts towards creating vaccines. Amongst the first to receive the vaccines were healthcare workers. This research focused on tracking the evolving COVID-19 epidemiology amongst healthcare workers (HCWs) in Galway University Hospital.

**Materials and Methods**

HCWs who were patient-facing and vaccinated were enrolled, with data collected from 45 participants from the first 11 weeks of the study used for this research. Participants completed an enrollment questionnaire, took a nasal swab and symptoms survey on the day of enrollment and every week after until study completion, and had their blood sample taken on enrollment and every 12 weeks after until study completion to track COVID-19 vaccine and infection titers.

**Results**

Most participants were fully vaccinated if not boosted with the Pfizer vaccine (≈90%). Swab results from week 3 had the greatest number of positives 14 (42%) followed by week 8, 5 (14%). Many cases were asymptomatic (57.6%). Infection history and blood test results showed a small percent who had no documented history of COVID-19 infection had COVID-19 previously (9.09%) as their serological samples read positive for nucleocapsid antibodies. There were those who had a documented history of COVID-19 infection but whose blood test results read negative for nucleocapsid antibodies (12.5%) indicating no previous infection.

**Conclusion**

Asymptomatic infection was present in many who tested positive highlighting the importance of continual mask wearing in the hospital despite vaccination. Moving forward considering whether COVID-19 antibodies will be protective, how asymptomatic infection presents, and the consequences of asymptomatic HCWs will be important.

**Disclosure**

None

## P7 Gastrointestinal motility in the very preterm infant and its implications for clinical care

### Colussi-Pelaez M.^1^, Dempsey E.^1,2,3^, Stanton C.^1,3^

#### ^1^ The School of Medicine UCC; ^2^ Cork University Maternity Hospital; ^3^APC Microbiome Ireland

*BMC Proceedings 2023*, **17(Suppl 4)**:P7

**Background**

Complete absorptive and electrical function of the gastrointestinal system develops late in gestation and consequently premature infants lack intestinal maturity. Hence, premature infants have slowed gastrointestinal motility compared to term infants, which hinders nutritional and growth outcomes. The primary objective is to establish the relationship between feeding and stooling behaviour in preterm infants <32 weeks gestation. The secondary objective is to explore other factors that influence stooling in this group.

**Materials and Methods**

This retrospective cohort study looked at a group of n=129 preterm infants <32 weeks gestation born at the Cork University Maternity Hospital (CUMH) between 2017 and 2021. Data was collected from the CUMH electronic record system and subsequently analysed using SPSS.

**Results**

Preterm infants with lower gestational ages stool less frequently in the first week of life compared to the second week (p=0.013). However, a univariate ANOVA, found that the primary feed type of maternal breast milk had the strongest influence over increased stooling frequency in the second week of life (p=0.009). There was also a positive correlation between preterm infants reaching full enteral feeds earlier and transitioning from meconium to normal stool earlier (p<0.001).

**Conclusion**

Our results suggest that feed advancement can influence earlier transition to normal stool. Associations like these, between feeding regimens received and stooling behaviour in the early weeks of life, can aid CUMH to identify patterns and optimize care of preterm neonates.

## P8 A feasibility study for the classification of Diabetic macular oedema from optical coherence tomography scans using deep learning

### Breathnach C.^1^, Harney F.^1,2^, Simpkin A.^3^, Hickey R.^1^, O’Keeffe D.^1,4^

#### ^1^School of Medicine, University of Galway, Galway, Ireland; ^2^Department of Ophthalmology, University Hospital Galway, Galway, Ireland; ^3^School of Mathematical and Statistical Sciences, University of Galway, Galway, Ireland; ^4^Department of Endocrinology, University Hospital Galway, Galway, Ireland

*BMC Proceedings 2023*, **17(Suppl 4)**:P8

**Background**

Diabetic Macular Oedema (DME) is a complication of poorly controlled Diabetes Mellitus that can threaten vision. DME is reliably detected using optical coherence tomography (OCT). Deep learning algorithms can be used to detect retinal pathology, including DME, from OCT scans.

**Materiald and Methods**

Anonymised OCT images were retrospectively obtained from 950 patients at University Hospital Galway. The images were taken at the foveal level and were graded by a consultant ophthalmologist to classify the level of DME present on a novel scale (Normal, DME not affecting the foveal contour and DME affecting the foveal contour). Other pathologies were excluded. A deep learning algorithm was validated using cross-validation, and then evaluated on an additional test dataset. The test set was graded by a second ophthalmologist for comparison.

**Results**

In detecting DME, the algorithm achieved an average accuracy of 94.34% on cross-validation and an accuracy of 92.47% on the test dataset, compared to the first ophthalmologist. The algorithm detected DME with an accuracy of 75.73% accuracy compared to the second ophthalmologist. When the detecting the DME class, the algorithm achieved an average accuracy of 89.34% on cross-validation and an accuracy of 86.61% on the test dataset compared to the first ophthalmologist. The algorithm detected the DME class with a 64.02% accuracy compared to the second ophthalmologist.

**Conclusion**

This study suggests promising results for the use of deep learning in the detection of DME in an Irish population. Refinement of the DME classification levels is required and further work could improve the algorithm accuracy.

**Disclosure**

Funding from Health Research Board of Ireland

## P9 Specialist palliative care and dementia: staff challenges and learning needs

### Currie S. J., Timmons S.

#### University College Cork

*BMC Proceedings 2023*, **17(Suppl 4)**:P9

**Background**

Dementia is an increasingly prevalent, life-limiting illness that warrants high-quality palliative care (PC). However, people with dementia (PwD) receive suboptimal PC. Inadequate staff education/training is a barrier to providing high-quality PC for PwD. This study aimed to explore specialist palliative care (SPC) teams’ challenges, learning needs, and preferred modes of education delivery related to dementia care.

**Materials and Methods**

This was a mixed-methods study involving a survey and one focus group. SPC staff were recruited through the Irish Palliative Medicine Consultants’ Association and via hospices in Cork, Waterford, Limerick, and Dublin. Survey data were collected from 76 staff on challenges, learning needs and preferred modes of education delivery. Quantitative analysis was descriptive and qualitative analysis from open-answer survey questions and one focus group was thematic.

**Results**

Based on data from 5-point Likert scales, the most challenging factors were: timely access to community agency support; timely access to specialist support; and managing the needs of PwD within the practice setting. Thematic analysis revealed challenges around the timing/duration of PC involvement, prognostication, and lack of knowledge of local services. Staff ranked their learning needs as highest in: non-pharmacological management of non-cognitive and cognitive symptoms; clinical differentiation of dementia subtypes; and pharmacological management of cognitive symptoms. 79.2% of staff preferred formal presentations by dementia-care specialists and 76.6% preferred e-learning access.

**Conclusions**

Several dementia-care challenges and learning needs have been identified by SPC staff. These may help inform the design and delivery of more tailored education programmes for SPC staff, with the ultimate goal of improving PC for PwD.

## P10 Female medical students’ perception of a career in orthopaedic surgery: a comparison study between Ireland and Sweden

### Doran C.^1^, Hennessy O.^2^

#### ^1^School of Medicine, University of Galway (UofG); ^2^Department of Trauma and Orthopaedic Surgery, Crumlin Hospital

*BMC Proceedings 2023*, **17(Suppl 4)**:P10

**Background**

Women account for half of medical school classes, yet there is a lack of female surgeons. Per the Irish trainees association, 10% of orthopaedic trainees are female, compared to 35% of trainees in Sweden. Aims: To investigate factors contributing to disproportionately low application rates to Orthopaedic Surgery (OS) by female students in Ireland and determining if attitude differences between a low female trainee % country (Ireland) and a high % country (Sweden) exist.

**Materials and Methods**

Questionnaire was e-mailed to medical students within University of Galway (UofG) and Uppsala University (UU). SPSS (version 27.0) was used. P values <0.05 were considered statistically significant.

**Results**

129 female medical students responded, 58.9% of participants originating from UofG and 41.1% originating from UU.

- Time to have children: 32.9% of Irish students said it would strongly discourage them from entering orthopaedics whereas 0% of Swedish students felt the same (P<.001).
- Taking maternity leave: 27.6% of Irish students said it would strongly discourage them from entering orthopaedics whereas 0% of Swedish students felt the same (P<.001).
- Family time: 42.1% of Irish students said it would strongly discourage them from entering orthopaedics whereas 3.8% of Swedish students felt the same (P<.001).
- Work/Life Balance: 26.3% of Irish students said it would discourage them from entering orthopaedics whereas 0% of Swedish students felt the same (P<.001).

**Conclusion**

The increased % of female trainees within Sweden can be attributed to the different perceptions amongst medical students. Targeting these issues will positively reorient Irish medical students’ perceptions of OS, thus increasing the number of female surgical trainees.

## P11 Paediatric head injuries and compliance with Paediatric Emergency Care Applied Research Network (PECARN) Guidelines in an inner-city hospital 2020

### Forde N., Murphy A.

#### University College Cork

*BMC Proceedings 2023*, **17(Suppl 4)**:P11

**Background**

To analyse the data and investigate if PECARN guidelines were met in relation to head injured children and report on the demographics of these patients.

**Materials and Methods**

Descriptive retrospective database study. Emergency Department (ED) chart review and IMPAX CT report review in Mercy University Hospital (MUH). Paediatric patients >1year and <=16 years presenting to ED in a 1-year period with a head injury, n= 201. All paediatrics that were triaged in 2020 as suffering from a head injury were collected on a Microsoft Excel Database and relevant variables were compiled. Those under 1 year and over 16 years were excluded. Variables collected include age, gender, mechanism of injury (MOI), date and time of presentation, arrival at ED, triage category, loss of consciousness (LOC) and vomiting status. ED files for patients with LOC, altered mental status (AMS) or vomiting at triage were interrogated. Further information was gathered via the IMPAX radiology system for CT brain requests. Data was transferred to SPSS for further statistical analysis.

**Results**

Of n=201, 42.3% were female (n=85) and 57.7% were male (n=116). The median age of presentation was 4 years (IQR 6). Those aged 1 years had the highest frequency of attendance at 18.9% (n=38), there was a negative association between number of presentations and age (r=-0.89, p<0.001). The most common MOI was a fall within the household (59.7%) followed by injuries playing outside the home (21.9%). There was no statistical difference between gender and MOI (Ï‡2 11.28, p=0.08). Of the n=201, n=25 presented with LOC, AMS and/or vomiting. Those that presented with LOC and/or vomiting were significantly more likely to be referred for CT (p-values<0.001). N=10 were deemed to warrant a CT for further investigations. Of these, n= 9 met the PECARN guidelines for a CT.

**Conclusion**

Head injuries are a common presentation to the MUH ED. Physicians generally follow PECARN guidelines. In 2020, one patient underwent CT who did not fulfil PECARN criteria.

## P12 Correlation of NT-proBNP and blood volume monitoring to assess volume status in maintenance haemodialysis patients

### Eftekhari A.^1^, Yousif I.^2^, Moran A.^2^, Dunne Ó.M.^1,2^

#### ^1^University of Galwa; ^2^Department of Nephrology Letterkenny University Hospital

*BMC Proceedings 2023*, **17(Suppl 4)**:P12

**Background**

Volume management in end-stage renal disease (ESRD) is important to reduce cardiovascular complications and mortality but the most appropriate tool to guide volume of fluid removal on haemodialysis (HD) is not known. Clinicians perform clinical assessments to decide on volume of fluid removal. Blood volume monitoring (BVM) is used during HD. NT-proBNP is also used to evaluate volume status. Our aim was to analyse measures of volume assessment in an incident maintenance HD population.

**Materials and Methods**

57 maintenance HD patients were included. Baseline patient characteristics and symptoms of volume overload were recorded. 2-week average volume of fluid removal per HD session was calculated. Occurrence of intradialytic hypotension was noted. NT-proBNP levels pre- and post-HD were measured. Relative blood volume (RBV) values pre- and post-HD were noted from the corresponding HD session. Echocardiogram results were reviewed to categorize patients with heart failure (HF) if EF <50%. Patients with ≥2 kg interdialytic gain were categorized as high fluid-gains. Changes in measurements before and after HD were calculated and compared using paired Student’s t-test, correlation measures and One-way ANOVA.

**Results**

No statistically significant correlation between NT-proBNP and RBV levels pre and post-HD. BVM measurements are moderately correlated with UF removed—Pearson correlation 0.492, p-value <0.0001. Although a small sample, one-way ANOVA post-hoc tests revealed that post-HD NT-proBNP was significantly higher (by 3270.22 pg/ml) in HF with high-fluid-gains, when compared to no-HF with high fluid-gains (p-value= 0.032, [241.7, 6298.74]).

**Conclusions**

BVM with clinical patient assessments should continue to guide fluid removal prescription. NT-proBNP may only be relevant in patients with HF.

## P13 A review of central nervous system lymphomas (CNSL) diagnosed at a single tertiary referral neuroscience centre

### Gupta E. M.

#### School of Medicine, University College Cork

*BMC Proceedings 2023*, **17(Suppl 4)**:P13

**Background**

Primary central nervous system lymphoma (PCNSL) is a rare form of non-Hodgkin lymphoma that develops within the brain and spinal cord. This study aims to identify the incidence, demographics and presentation of those diagnosed with PCNSL in Cork University Hospital over a 10 year period, and examine the pathology and outcomes of these patients.

**Materials and Methods**

This is a retrospective chart review. Neuropathology records identified 74 patients with a diagnosis of PCNSL from January 2011 to December 2020. The relevant information of the patients was collected. Descriptive data analysis was conducted and Kaplan-Meier survival analysis examined overall survival.

**Results**

There were 50 patients with PCNSL identified from 2011 to 2020, which equated to an incidence rate of 0.417 per 100,000. The other 24 patients had systemic lymphoma that metastasised. Mean age at diagnosis was 63.9 years. Confusion, ataxia and headache were the commonest presenting symptoms. Ninety-eight percent were classified as diffuse large B-cell lymphoma (n=49). One patient had immunodeficiency-associated lymphoma. The most common radiological sites of lesion was supratentorial (n=36). All patients tested positive for CD20 (100%). At present, 17 patients are alive, 25 are dead from PCNSL, 7 are dead from other causes, while 1 patient’s outcome is unknown. Mean survival time was 41.7 months overall. It was 33.3 months in the female population and 45.2 months in the male population.

**Conclusion**

This is the second study to examine PCNSL in Ireland, and the first to report referrals to this tertiary neuroscience centre. The data suggests the survival of patients may be improving.

## P14 Prognostic factors for mortality in bronchiectasis patients: a literature review

### Kon A. M. H.^1,2^, Ng S. H. X.^3^, Chai G. T.^4^, Lee K. C.^5^, Pradeep P. G.^3^, Tan W. S.^3^, Hum A.^6^

#### ^1^University of Galway; ^2^School of Medicine, University of Ireland, Galway, Ireland; ^3^Health Services and Outcomes Research, National Healthcare Group, 3 Fusionopolis Link, #03-08, Singapore 138543; ^4^Department of Respiratory and Critical Care Medicine, Tan Tock Seng Hospital, 11 Jalan Tan Tock Seng, Singapore 308433; ^5^School of Medicine, Nanyang Technological University, 11 Mandalay Road, Singapore 308232; ^6^Department of Palliative Medicine, Tan Tock Seng Hospital, 11 Jalan Tan Tock Seng, Singapore 308433, The Palliative Care Centre for Excellence in Research and Education, Dover Park Hospice, 10 Jalan Tan Tock Seng, Singapore 308436

*BMC Proceedings 2023*, **17(Suppl 4)**:P14

**Background**

Non-cystic fibrosis (CF) bronchiectasis is a chronic, debilitating lung condition and may result in an increased mortality. The ability to predict deterioration and subsequent mortality in these patients is vital in clinical decisions and treatment plans. However, the lack of clarity in its pathogenesis renders accurate prognostication challenging, and there is currently no comprehensive review of prognostic factors for bronchiectasis. We aim to provide a descriptive overview of the known prognostic factors and prognostication tools for mortality in bronchiectasis patients, to better understand the current research landscape and gaps in knowledge.

**Materials and Methods**

We conducted a literature review of studies published between 2000 and 2020 on the prognostic factors associated with mortality in non-CF bronchiectasis.

**Results**

We identified 27 studies, with 18 studies predicting mortality beyond 5 years. Prognostic factors identified were classified into nine major themes: socio-demographic factors, general health status, admission outcomes and symptoms, biomarkers, microbial infection, lung disease severity measures, respiratory comorbidities, prediction tools and treatment. General health status, lung disease severity measures and socio-demographics were most frequently assessed. Disease severity and respiratory comorbidities were most commonly associated with poorer survival (disease severity: 67% of models; respiratory comorbidities: 56%)

**Conclusion**

Our review provides an overview of prognostic factors in bronchiectasis. Prognostic efforts should focus on validating existing tools, and incorporating respiratory comorbidity into these models. Further work will entail a detailed listing of individual prognostic factors and appraisal of study quality. Accurate prognostication will strengthen our understanding of bronchiectasis patients and their needs for clinical decision-making.

## P15 A study of severe maternal morbidity and critical care requirements in pregnancy using the NPEC severe maternal morbidity audit data in Ireland

### Kaskun O.^1^, Leitao S.^2^

#### ^1^University College Cork; ^2^National Perinatal Epidemiology Centre

*BMC Proceedings 2023*, **17(Suppl 4)**:P15

**Background**

Maternal health is a measure of socioeconomic progress. Assessing severe maternal morbidity (SMM) is an important quality indicator. The National Perinatal Epidemiology Centre (NPEC) conducts yearly audits in Ireland to track SMM.

**Materials and Methods**

To study the characteristics of women who experienced SMM in Ireland between 2011-19, and to analyse the prevalence of 16 specific SMMs and potential associated factors. A secondary analysis of audit data collected by NPEC from 19 Irish Maternity Units between 2011-19 was performed. Units provided anonymised standardised demographic and clinical data from eligible women (recently pregnant women up to 42 days following pregnancy end). Descriptive analysis and correlation testing was performed.

**Results**

3093 SMM cases were identified. Two in five cases were in women aged 35 or older (42.0%) and nulliparous (41.9%). Almost a quarter of cases (24.3%) occurred in women with obesity. SMM rate increased by 68% between 2011-19 from 3.85 to 6.47 per 1,000 maternities. Major obstetric haemorrhage (MOH) accounted for half of SMM cases (50.2%), and intensive or coronary care unit admission was reported for two in five SMM cases (43.4%). Higher level of critical care was associated with women aged 30 or older (p=0.021).

**Conclusions**

SMM affects many women and its incidence increased in the nine years of the audit. Further study is required to reduce the high occurrence of MOH. Increased BMI and age are risk factors for SMM, recommending closer monitoring. Improved data collection is needed to establish the influence of smoking and alcohol consumption on SMM incidence.

## P16 Investigation of the function of adpS within Staphylococcus epidermidis biofilm formation and medical device-related infections

### Mikasauskaite D.^1^, Burke O.^2^, Zeden M.^2^, O'Gara J.^2^

#### ^1^University of Galway; ^2^Microbiology Department, University of Galway

*BMC Proceedings 2023*, **17(Suppl 4)**:P16

**Background**

Staphylococcus epidermidis is an opportunistic pathogen, frequently implicated in nosocomial infections. S. epidermidis can form biofilms which protect it against the host immune system and antibiotics. The 291 bp adpS gene (autoinducer degrading protein of Staphylococcus) was identified in a biofilm negative mutant. Increased biofilm production from overexpressing adpS implicated a potential role for AdpS in S. epidermidis biofilms. The aim of this project is to investigate the function of AdpS through the identification of protein interaction partners to aid research into treatments for antibiotic resistant biofilm-based infections.

**Materials and Methods**

In searching for protein binding partners of AdpS, by combining a Bacterial Two Hybrid system and genomic DNA library screening approach using a clinical S. epidermidis strain CSF41498 isolated from a device-related infection, this project identified 10 protein interaction partners of AdpS. Positive partners were identified using Sanger sequencing and bioinformatics.

**Results**

Among the interacting partners of AdpS identified were a LytR family transcriptional regulator, a non-ribosomal peptide synthase, a YSIRK domain containing protein, protein translocase subunit SecA1, a cytochrome C assembly protein and a phosphonate ABC transporter.

**Conclusion**

The partners identified provide indications of the function of AdpS. Its interaction with the LytR family transcriptional regulator, part of the LytR/LytS system that regulates biofilm formation in Staphylococcus aureus, is an interesting candidate to investigate further. The protein-protein interactions identified provide a basis for future works to assign a more accurate character profile to this cryptic gene, with the hope of providing insight for therapeutic intervention for biofilm-originated infections.

## P17 Unclassified bleeding disorders - a review of diagnosis and treatment in a Comprehensive Coagulation Centre

### Kemp S.^1^, Crowley, M.P.^2^, Duggan C.^2^

#### ^1^University College Cork; ^2^Comprehensive Coagulation Centre, CUH

*BMC Proceedings 2023*, **17(Suppl 4)**:P17

**Background**

Recent research has demonstrated that the number of patients with unclassified bleeding disorders (UBD) is growing. As a diagnosis of exclusion, UBD describes a heterogenous group with diverse clinical presentations. This complicates standardization of diagnosis and management. Evidence suggests that prophylaxis with tranexamic acid (TxA) Â± desmopressin is effective in managing these patients. However, only three studies have studied this.

This study aimed to examine clinical presentation, diagnostic work-up and treatment outcomes in UBD.

**Materials and Methods**

A retrospective chart review of all UBD patients (n=48) attending the Comprehensive Coagulation Centre, CUH from January 2000-July 2021 was performed. Electronic chart data was examined for patient demographics, bleeding symptoms, and transfusion history. Laboratory investigations were assessed under four headings: FBC and blood film, vWF screen, platelet function, and clotting factor levels. Prophylaxis for invasive procedures was examined, and outcomes recorded. Results were compiled and analysed on Microsoft Excel®.

**Results**

In our cohort, 92% were women and mean age of presentation was 43. The most common symptoms were menorrhagia (68%) and cutaneous bleeding (65%). 22 patients required transfusion for their symptoms. All patients had an FBC, vWF screen, and platelet function assay performed. Only 14 (29.2%) patients had a documented blood film and 10 (20.8%) had a full clotting factor assay. Seventy-five invasive procedures were treated with TxA ± desmopressin, with 94.7% achieving successful haemostasis.

**Conclusion**

Diagnosis of UBD could benefit from a clearly defined protocol for investigations. TxA proved effective in symptom control. Further research is necessary to achieve standardization of patient diagnosis and management.

## P18 The experiences and perceptions of Donegal general practitioners with the primary prevention, acute presentation and post-discharge care of their patients with strokes and transient ischaemic attacks

### O'Connell C.^1^, Memon A.^2^

#### ^1^University of Galway; ^2^Acute Stroke Unit, Letterkenny General Hospital

*BMC Proceedings 2023*, **17(Suppl 4)**:P18

**Background**

To understand Donegal GPs approach to primary prevention, acute management and subsequent post-discharge care of their patients who suffer Strokes and Transient Ischaemic Attacks (TIAs).

To investigate local GPs interactions and satisfaction with the LUH Acute Stroke Unit.

**Materials and Methods**

An anonymous questionnaire was disseminated electronically to 34 practices across County Donegal between June and August 2022.

**Results**

19 GPs from 18 different practices responded. When asked about acute presentations to GP, 88.5%(n=15) of respondents felt confident recognising and diagnosing a potential stroke, and 93.1%(n=16) a TIA. 58.8%(n=10) believed GPs receive suitable training and guidance in recognition and management of acute stroke and 52.9%(n=9) for TIAs.

As for the primary prevention of stroke, 94.7%(n=17) of GPs engage in opportunistic screening for atrial fibrillation and 58.8%(n=10) use scoring systems for evaluating risk. However, only 38.9%(n=7) of GPs refer their patients to specialist clinics for control of risk factors relevant to stroke.

52.7%(n=10) of GPs expressed concerns around their patient’s compliance with recommended pharmacological interventions post-stroke, and 68.4%(n=13) with recommended lifestyle interventions.

**Conclusion**

The majority were confident in recognising stroke and TIA signs and symptoms, but no consensus was reached on whether the training and guidance in this area was satisfactory. There is scope for encouraging greater use of scoring systems in stratifying risk, alongside encouraging more referrals to specialist Stroke Clinics. A large proportion of the respondents had concerns around their patient’s compliance with secondary prevention measures.

## P19 Medical record quality in Ireland; an audit of clinical record governance in ireland conducted in the Cork University Hospital in 2022

### Mooney E.^1^, Roe C.^2^

#### ^1^University College Cork; ^2^Quality Department, Cork University Hospital

*BMC Proceedings 2023*, **17(Suppl 4)**:P19

**Background**

Medical documentation should be clear, accurate and complete. Standards exist to uphold how information is recorded in a patient's personal medical record although failures frequently occur in meeting these requirements. The audit being presented hoped to examine where errors do commonly occur to inform future projects to improve medical record quality.

**Methods**

In February 2022 in Cork University Hospital a thorough analysis for absent or incorrect documentation in 30 medical records was conducted and compared against the compulsory standards outlined through the HSE. A number of repeated breaches of protocol were then identified. Over the 11 specific questions being asked across 6 separate domains , a mean score of 54% adherence to the required standards was found. The lowest adherence rate per single standard was found to be just 3% compliance.

**Results and Conclusions**

From these initial results, we can conclude that there must be more education and stricter enforcement of medical documentation quality and further exploration as to what causes might be to blame. This audit may now inform future interventions and quality improvement projects.

## P20 A longitudinal evaluation of the impact of the COVID-19 pandemic on patients with pre-existing mood disorders

### O'Gorman E.^1^, Rainford A.^1^, McLoughlin J.^2^, Delaney E.^1^, Hallahan B.^1,2^

#### ^1^School of Medicine, University of Galway; ^2^University Hospital Galway

*BMC Proceedings 2023*, **17(Suppl 4)**:P20

**Background**

The WHO’s declaration of the COVID-19 pandemic precipitated national lockdowns and reduced availability of therapeutic activities for individuals attending mental health services. Initial data demonstrated deleterious impact for individuals with pre-existing mood disorders. We aimed to assess longitudinally the psychological, social and functional impact of the COVID-19 pandemic on individuals attending a secondary mental health service with either bipolar disorder or Emotionally Unstable Personality Disorder (EUPD).

**Materials and Methods**

Study participants (n=36) were contacted by telephone for semi-structured interviews (n=29) to attain repeat data relating to several clinical and demographic variables, approximately 24 and 12 months after initial collection. The same validated psychometric instruments were employed with free-text data collected to ascertain the impact of the COVID-19 pandemic. Paired t-tests and repeated measures analysis of co-variance (Wilkes-Lambda statistic) were utilised to compare psychometric data between disorders and between baseline and both follow-up visits.

**Results**

Individuals with EUPD had significantly greater anxiety (t=4.45,p<0.001) and depressive (t=2.52,p=0.020) symptoms, and greater hopelessness (t=2.67,p=0.014) compared to individuals with bipolar disorder at this longitudinal review. No significant difference over time was noted for anxiety, depressive or impulsivity symptoms or hopelessness (p>0.05). Qualitative data demonstrates positivity regarding individuals’ future and views that mental health services can implement changes such as increased use of telepsychiatry in the future.

**Conclusion**

Individuals with EUPD demonstrate significantly greater symptomatology compared to individuals with bipolar disorder. Despite positive views expressed, the provision of therapeutic interventions for individuals with EUPD is warranted given significant distress and symptoms being experienced.

## P21 Knowledge and attitudes toward alcohol use during pregnancy

### Pinter A.^1^, Gibson L.^1,2^ Glynn H.^3^

#### ^1^University College Cork; ^2^Paediatrics and Child Health, CUH; ^3^Obstetrics and Gynaecology, CUH

*BMC Proceedings 2023*, **17(Suppl 4)**:P21

**Background**

Foetal alcohol syndrome (FAS) encompasses neonatal presentations of cognitive, physical and behavioural abnormalities associated with the consumption of alcohol in pregnancy. Globally, FAS is one of the most preventable birth defects however, despite the known dangers, alcohol consumption remains alarmingly high. Specifically, in Ireland as many as 45.9% of women disregard guidelines to abstain. In order to develop prevention strategies, the level of knowledge regarding the safety of alcohol must be elucidated.

**Materials and Methods**

This study aimed to illustrate the current understanding of and attitudes towards alcohol consumption during pregnancy in women in Cork. Participants over 18 years of age with confirmed pregnancy were recruited through convenience sampling from the antenatal clinic at CUMH between October 2021 and March 2022.

**Results**

Of the 314 respondents, 13.1% continued to drink throughout their pregnancy. A majority (69.9%) disagreed with the statement that drinking a small amount of alcohol in pregnancy is safe and indicated that drinking alcohol may cause harm if at least one unit per day is consumed (80.1%). With regard to FAS knowledge, 62.6% indicated that they had heard of the condition, either through education (32%) or from the media (32.4%). Of those who had heard of FAS, 37.6% correctly identified its congenital defects, cause and duration.

**Conclusion**

Although a majority of women abstain from alcohol during pregnancy, the level of understanding surrounding the harmful impact of alcohol consumption during pregnancy may be inadequate for proper prevention. Further investigation is required to establish robust, community-based educational interventions by healthcare professionals in the future.

## P22 A retrospective review of patients with primary appendiceal neoplasms and the best practice guidance for the management of these tumours

### Ramanathan T.^1^, Choy M.^2^, O Connell L.^2^, Regan M.^2^, Joyce M.^2^, Nugent E.^2^

#### ^1^University of Galway, Ireland; ^2^Department of Colorectal Surgery, Galway University Hospital

*BMC Proceedings 2023*, **17(Suppl 4)**:P22

**Background**

Appendiceal neoplasms are a heterogeneous group of tumours that exhibit varying malignant potential. Multidisciplinary teams (MDTs) often have a large role to play in the discussion and management of individual patient care. This research aimed to conduct an evaluation of the surgical outcomes of patients at our institute with appendiceal neoplasms and explore the best practice guidance in each case.

**Materials and Methods**

A retrospective review of a gastrointestinal database in our institution was conducted from 2018 -2022. Patients with histologically confirmed neoplasms of the appendix were identified and secondary appendiceal tumours were excluded from analysis. Data collected included clinicopathological characteristics, incidence, and management, including referral to the national Neuroendocrine Tumour (NET) MDT or for consideration of Cytoreductive Surgery and Intraperitoneal Chemotherapy (CRS-HIPEC).

**Results**

A total of 32 patients were diagnosed with primary appendiceal neoplasms over the study period (n=32). The incidence rate was 0.17 per 100,000 of the population. Of the total cohort, 5 were aged <18 years at time of index surgery. Pseudomyxoma peritonei was the presenting complaint in two patients. 71% (n=23) were treated with appendicectomy or caecectomy as their index surgical procedure. Five patients were referred to the national NET MDT, of which three were paediatric patients. A further four patients were referred to The Mater Hospital for consideration of CRS-HIPEC.

**Conclusion**

Most patients with benign appendix neoplasms may be effectively managed via the local MDT. Referral to the national NET MDT or CRS-HIPEC unit should be considered for paediatric patients or those presenting with advanced disease.

## P23 Variation in the practice of wide local excision for melanoma in Ireland and the UK

### O’Connor E.^1^, O' Connell G.^2^ O' Connor C.^2^, Peach H.^3^, O' Shea S.^2^

#### ^1^University College Cork; ^2^South Infirmary Victoria University Hospital; ^3^Leeds Teaching Hospitals NHS Trust

*BMC Proceedings 2023*, **17(Suppl 4)**:P23

**Background**

Wide local excision (WLE) is gold standard practice in primary melanoma management, however, no national guidelines exist regarding its technique. The aims of our study were: to assess variation in the practice of WLE and to explore the effect of clinician’s specialty and grade.

**Materials and Methods**

This was an international, anonymised, cross-sectional study. An online questionnaire was distributed to IAD, BAD, Melanoma Focus, BAPRAS and BioGenoMEL members. Descriptive statistics were used. Secondary analysis, using Chi-squared test, was performed to examine the association between clinician demographics and technique.

**Results**

There were 128 respondents (response rate 3%): 57% dermatologists and 38% plastic surgeons. 80% were consultants. 44% had worked in their role for 3-10 years. 21% mentioned textbooks/media as part of WLE training, with most “learning on the job”. There was significant variation in planning and performing WLE. 59% considered margins already achieved; 71% marked margins with the skin relaxed. For 1cm WLE, 84% delineated 1cm from the scar edge; the remainder marked from the scar midpoint. Plastic surgeons preferred the latter (p=0.003). The majority orientated the primary and WLE in a longitudinal/oblique axis on the limbs, 83% and 81%, respectively. Only 40% sent dog ears for histology. 71% incised along the marked line; 27% incised outside it. 79% excised to deep fascia; 19% to the next biological margin.

**Conclusion**

This study demonstrates variation among clinicians performing WLE. We postulate that this could impact patient outcomes. A consensus statement is being developed, to achieve more consistency in the practice of WLE.

## P24 Wearable technology for patient orientated management of knee osteoarthritis

### Nugent A., Newell M., Duke C.

#### University of Galway

*BMC Proceedings 2023*, **17(Suppl 4)**:P24

**Background**

Functional Movement Assessment is critical especially in early-to-moderate Osteoarthritis. Wearable sensor technology could provide a more efficient solution for patient orientated assessment and monitoring of joint movements. The aim of this project was to explore the feasibility of using small wearable sensors to evaluate knee functional movement tasks of everyday activities.

**Materials and Methods**

A cross-section observational design was used. A convenience sample approach recruited 20 participants. Existing self-reported lower limb issues were recorded. Participants completed three functional movement tasks of everyday activities (walking, lunging and sit-to-stand) wearing lightweight sensors attached above and below the knee joint. Ethical approval was granted for this project by Galway University Hospital Ethics Committee.

**Results**

Twenty participants (16 females and 4 males), mean age 41 years participated in this study.

Initial examination of the raw collected data revealed an issue with transfer of accelerometer and gyroscope data from Bluetooth sensors to the smartphone. The expectation, based on the manufacturer specifications, was that both accelerometer and gyroscope readings were being collected at a rate of 50 Hz. This is the minimum frequency required for accurate generation of kinematic profiles using Seel's algorithm. However, the actual rate was approximately 35 Hz for accelerometer and 20 Hz for gyroscope.

Thus the reliability of the values obtained cannot be ascertained without further comparison using data collected in parallel with an independent fixed camera system.

**Conclusion**

Refinement of the current sensors and data downloading issues are needed for establishing reference values for dynamic knee joint performance in everyday activities.

## P25 A retrospective comparative evaluation of the factors associated with the decision to proceed with bariatric surgery intervention following initial consultation

### O'Flynn B., O'Boyle C.

#### University College Cork

*BMC Proceedings 2023*, **17(Suppl 4)**:P25

**Background**

With the increase in the obesity epidemic, bariatric surgery rates have escalated exponentially. Despite this, only a fraction of those referred for Bariatric consultation follow through with surgery. The aim of this project is the evaluation of the most frequent variables in people who attend bariatric surgical consultation and decline bariatric surgery in comparison with those who follow through with surgery.

**Materials and Methods**

The following parameters were recorded - age, sex, BMI, health insurance, diabetes, sleep apnoea, hyperlipidaemia, and psychological assessment tools.

Between January 2017 and January 2021, 234 patients attended an initial bariatric consultation at the Bons Secours bariatric service and completed pre-consultation questionnaires.

**Results**

There were 128 (74%) female and 44 (26%) male patients for consultation. 93 (54%) underwent subsequent surgery and the remainder were lost to follow-up. Unsurprisingly, private insurance cover had a significant influence on whether patients proceeded with surgery 81(80%) insured in comparison to 13(12%) uninsured, p <0.01, Pearson Chi square test result = 3.7881, P = 0.05. The overall BDI and HAD scores for the group were in the moderately depressed range. However, patients not proceeding with surgery had a significantly higher BDI and HAD score than those who ultimately proceeded to surgery

**Conclusion**

Patients who decline subsequent bariatric surgical intervention had statically significant higher BDI and HAD scores on completing questionnaires prior to their initial consultation. Insurance was also associated with an increased likelihood of surgery. This investigation highlights the need for bariatric surgery to be made more widely available.

## P26 Characterisation of potential new formulations for the treatment of Diabetic macular oedema

### Walsh A., Duffy G.P., O'Dwyer J.

#### ^1^Discipline of Anatomy, School of Medicine, Ollscoil na Gaillimhe - University of Galway, Ireland

*BMC Proceedings 2023*, **17(Suppl 4)**:P26

**Background**

Diabetic macular oedema (DME) is the leading cause of blindness in the working age population. DME occurs due to the formation of aberrant blood vessels in the retina resulting in oedematous swelling. One challenge in developing more effective treatments is the absence of accurate in-vitro models of DME. This study aimed to test potential future treatments in different in-vitro systems to assess the potential efficacy and injectability of the treatments and to determine the variation between in-vitro test systems.

**Materials and Methods**

2x10^5 retinal pigment epithelial cells (ARPE-19) were plated on Transwell membranes for 18 days until monolayer formation occurred. Monolayer integrity was measured using fluorescently tagged dextran. Porcine retinas were dissected and retinal integrity was investigated using light microscopy and by placing the retinas on a Franz diffusion apparatus. Formulations were tested on both the cell monolayer and porcine retina. Injectability was determined using porcine eyes.

**Results**

The ARPE-19 monolayer formed a viable membrane suitable to test selected formulations. The same trend was observed in results from the porcine retina as with the ARPE-19 monolayer when measuring the desired outputs. Selected formulations could be injected into the porcine eye via a human syringe operator and formulation remained present post-injection.

**Conclusion**

This study indicates that a cultured ARPE-19 cell monolayer grown on Transwell membranes is an alternative to using fresh retinas to test formulations. A human syringe operator can inject tested formulations into the eye, which is critical for clinical translation.

## P27 Combined EEG focal slowing with phase reversal: assessing diagnostic implications

### Rokos A.^1^, Antonio dos Santos Leite C.^2^, O’Sullivan S.^2^

#### ^1^University College Cork; ^2^Bons Secours Cork

*BMC Proceedings 2023*, **17(Suppl 4)**:P27

**Background**

Electroencephalography (EEG) is commonly combined with the medical history to diagnose and assess a wide variety of neurological disorders. Unfortunately, misdiagnosis following EEG is not uncommon and the effects can be extremely detrimental to an individual’s quality of life. A novel EEG marker of combined focal-slowing with phase-reversal has recently been observed and is of unknown clinical significance.

**Materials and Methods**

This study assesses whether EEG with combined focal-slowing and phase-reversal is associated with specific demographic or clinical factors. A retrospective chart, EEG, and physician letter review was conducted on patients over the age of 18, referred to Bon Secours Cork for EEG (2019-2022), and had a salient EEG finding of Focal-Slowing (n = 30). Eligible patients were grouped into either a case group, showing combined focal-slowing and phase-reversal (n=16), or a control group, showing traditional focal-slowing without phase-reversal (n=14). Ï‡-squared and t-tests were used to compare the groups in terms of demographic variables, treatments, and diagnostic outcomes.

**Results**

The findings demonstrated that the presence of focal-slowing with phase-reversal is very strongly associated with seizure type disorder diagnosis (p = 0.011, X2 = 6.467, Phi and Cramer’s V = 0.464). No significant association was identified between forcal slowing with phase reversal and petient demographics.

**Conclusions**

This is the first study to investigate the clinical implications of focal slowing with phase reversal and these data may help inform clinicians regarding the interpretation of the presence of this novel marker in future EEG analyses.

## P28 Moderate intensity aerobic exercise increases plasma ketone concentrations in healthy female adults

### Walsh É.^1^, Alshogairi A.^1^, Lynch C.^1,2^, Kelly T.^1^, O'Brien T.^1,2,3^, Leahy M.^4^, Finucane F.M.^1,2,3^

#### ^1^School of Medicine, CMNHS, University of Galway, Ireland; ^2^Bariatric Medicine Service, Centre for Diabetes, Endocrinology and Metabolism, Galway University Hospitals, Galway, Ireland; ^3^HRB Clinical Research Facility, University of Galway; ^4^School of Physics, University of Galway, Ireland

*BMC Proceedings 2023*, **17(Suppl 4)**:P28

**Background**

With the emerging scientific interest in the role of physiological ketosis in human health, a better understanding of the factors influencing plasma ketone concentrations and ketogenesis is required. We sought to describe the influence of aerobic exercise on fasting beta-hydroxybutyrate (BHB) in healthy female adults.

**Materials and Methods**

We conducted a single centre cross-over study of healthy female adults who had BHB concentrations measured continuously over three hours after fasting and again after a 60-minute bout of moderate intensity aerobic exercise at 70% of their estimated maximal heart rate. BHB was measured with the Abbott® GlucoMen Areo 2K device and differences in BHB over time were assessed with the paired t-test.

**Results**

We recruited 10 participants with a mean age of 26.3±8.1 years. Compared to fasting, BHB levels were statistically significantly higher from 30 minutes into the exercise bout, at 0.45±0.16 versus 0.3±0.12 mmol/l, respectively (p=0.026) with differences peaking within the 60 minutes, but not persisting to 90 minutes after completion of the exercise bout.

**Conclusion**

Moderate intensity aerobic exercise increases plasma BHB levels compared to fasting alone, within 30 minutes of starting exercise, and with elevations in ketone levels up to one hour after exercise stops. Whether different modalities, duration and intensity of exercise influence ketone concentrations, and the relevance of this to metabolic health, remains to be determined.

## P29 An evaluation of mixed-reality enhanced clinical teaching of medical students - a qualitative study of learners’ perspectives

### Volz J.^1^, Connolly M.^2^, O'Brien N.2, Iohom G.^2^

#### ^1^University College Cork; ^2^CUH Department of Anaesthesiology and Intensive Care

*BMC Proceedings 2023*, **17(Suppl 4)**:P29

**Background**

Augmented reality (AR) is a virtual environment that allows users to interact with physical and virtual elements in real-time.[1] One type of augmented reality tool, head-mounted devices (HMDs), have been used in medical training at several stages, with positive learner response.[2-4] Recent studies have evaluated a commercially available HMD, the Microsoft HoloLens 2, in roles such as the remote broadcasting of live ward rounds and bedside teachings.[5, 6] These studies have indicated that clinical education via the HoloLens 2 is feasible but did not use the mixed-reality facility of the headset and have not examined the learning efficacy of the sessions. Additionally, the evaluation of this technology-enhanced learning modality has proved challenging due to a lack of empirical research data and validated evaluation strategies.[2, 7, 8]

**Materials and Methods**

This qualitative study assesses the Microsoft HoloLens in two separate applications to investigate its value as a teaching tool. We first evaluated its use in remote delivery of bedside tutorials on anaesthetic preoperative history and airway examination sessions during the COVID-19 pandemic. Second, we explore a novel proof of concept use of the technology as an adjunct for procedural skills acquisition. We evaluated the outcomes using validated Likert scales, open-ended qualitative response elements, key-informant interviews, and a ‘think-aloud’ method of collecting real-time feedback during procedural skills acquisition.

**Results and Conclusions**

The summation of this data suggests that learners view the Microsoft HoloLens2 enhanced learning favourably. Participants found this method easily usable and effective as traditional teaching techniques and offered support for its inclusion in medical school curricula.

## P30 Identifying HIV stigma and discrimination in healthcare settings in Ireland from health care workers’ perspectives

### Denierio M.^1^, Vaughan E.^2^

#### ^1^School of Medicine, University of Galway; ^2^Health Promotion and Research Centre, University of Galway

*BMC Proceedings 2023*, **17(Suppl 4)**:P30

**Background**

Stigma for people living with HIV (PLHIV) still permeates healthcare, creating barriers to accessible care, treatment, and testing. These stigmatising practices occur on individual and institutional levels, with an inadequate response in policy, training and education on HIV. This research aims to identify stigmatising practices by healthcare workers, enacted and observed towards PLHIV, and to identify gaps in knowledge and policy that facilitate these practices.

**Materials and Methods**

Quantitative and qualitative survey data was compiled from different healthcare sectors. Survey items were drawn from questions and comments expressed in stakeholder panels on training, primary and secondary stigmatising behaviours, knowledge regarding viral load, awareness of advocacy campaigns, and attitudes toward at-risk populations.

**Results**

There were significant findings in stigmatising behaviours toward PLHIV in healthcare workers (n=298). Of behavioural interest were fears of dressing wounds (21%), taking bloods (28%), and observed stigma from medical professionals speaking badly about PLHIV (20%). 57% of respondents were unaware if their facility had protective guidelines for PLHIV. 17% of healthcare workers were not aware of advocacy campaigns and significant gaps in knowledge of viral transmission and advocacy were identified. Lack of training was noted, 20% had undergone HIV training, 73% had undergone confidentiality training and 30% had undergone training to destigmatise at-risk populations.

**Conclusions**

The results identify gaps in knowledge in healthcare workers and misunderstandings of undetectable viral loads, transmission, and beliefs about at-risk populations. Combined with collaborations from PLHIV, this creates a framework for policies in training, education and advocacy to foster respect, inclusivity, accessibility and healthcare equality for PLHIV.

